# Chronic hepatitis B: dynamic change in Traditional Chinese Medicine syndrome by dynamic network biomarkers

**DOI:** 10.1186/s13020-019-0275-4

**Published:** 2019-11-21

**Authors:** Yiyu Lu, Zhaoyuan Fang, Tao Zeng, Meiyi Li, Qilong Chen, Hui Zhang, Qianmei Zhou, Yiyang Hu, Luonan Chen, Shibing Su

**Affiliations:** 1 Institute of Interdisciplinary Integrative Medicine Research, Shanghai University of Traditional Chinese Medicine, Shanghai, 201203 China; 2 Key Laboratory of Systems Biology, CAS Center for Excellence in Molecular Cell Science, Institute of Biochemistry and Cell Biology, Shanghai Institute of Biological Sciences, Chinese Academy of Sciences, Shanghai, 200031 China; 3 CAS Center for Excellence in Animal Evolution and Genetics, Chinese Academy of Sciences, Kunming, 650223 China; 4 Institute of Liver Disease, Shuguang Hospital, Shanghai University of Traditional Chinese Medicine, Shanghai, 201203 China; 5 Minhang Branch, Zhongshan Hospital, Fudan University/Institute of Fudan-Minhang Academic Health System, Minhang Hospital, Fudan University, Shanghai, 201199 China

**Keywords:** Disease progression, Dynamic network biomarkers, Chronic hepatitis B, Systems biology, Traditional Chinese Medicine syndrome

## Abstract

**Background:**

In traditional Chinese medicine (TCM) clinical practice, TCM syndromes help to understand human homeostasis and guide individualized treatment. However, the TCM syndrome changes with disease progression, of which the scientific basis and mechanism remain unclear.

**Methods:**

To demonstrate the underlying mechanism of dynamic changes in the TCM syndrome, we applied a dynamic network biomarker (DNB) algorithm to obtain the DNBs of changes in the TCM syndrome, based on the transcriptomic data of patients with chronic hepatitis B and typical TCM syndromes, including healthy controls and patients with liver-gallbladder dampness-heat syndrome (LGDHS), liver-depression spleen-deficiency syndrome (LDSDS), and liver-kidney yin-deficiency syndrome (LKYDS). The DNB model exploits collective fluctuations and correlations of the observed genes, then diagnoses the critical state.

**Results:**

Our results showed that the DNBs of TCM syndromes were comprised of 52 genes and the tipping point occurred at the LDSDS stage. Meanwhile, there were numerous differentially expressed genes between LGDHS and LKYDS, which highlighted the drastic changes before and after the tipping point, implying the 52 DNBs could serve as early-warning signals of the upcoming change in the TCM syndrome. Next, we validated DNBs by cytokine profiling and isobaric tags for relative and absolute quantitation (iTRAQ). The results showed that PLG (plasminogen) and coagulation factor XII (F12) were significantly expressed during the progression of TCM syndrome from LGDHS to LKYDS.

**Conclusions:**

This study provides a scientific understanding of changes in the TCM syndrome. During this process, the cytokine system was involved all the time. The DNBs PLG and F12 were confirmed to significantly change during TCM-syndrome progression and indicated a potential value of DNBs in auxiliary diagnosis of TCM syndrome in CHB.

*Trial registration* Identifier: NCT03189992. Registered on June 4, 2017. Retrospectively registered (http://www.clinicaltrials.gov)

## Background

Traditional Chinese Medicine (TCM) plays an important role in alternative health care. Nowadays, TCM is becoming more popular [1], because of increasing evidence of its efficacy [2–4]. Chronic hepatitis B (CHB) infection continues to be a global health problem. There are more than 400 million people worldwide affected with CHB, ranging from hepatitis B-virus (HBV) carrier, CHB, cirrhosis, and hepatocellular carcinoma [5]. Treatment of CHB is unsatisfactory and further progress is needed. However, TCM treatments for HBV-related diseases, especially when combined with Western medicine, have promising effects [6, 7]. TCM syndrome (“ZHENG” in Mandarin Chinese) is a key principle in TCM. Patients with the same disease would be treated differently by TCM practitioners, per TCM syndrome. Previous studies have shown that patients with different TCM syndromes displayed various responses after being treated with the same therapy [8, 9]. Furthermore, applying TCM without any consideration of the TCM-syndrome classification could cause serious side effects [10]. Liver-gallbladder dampness-heat syndrome (LGDHS), liver-depression spleen-deficiency syndrome (LDSDS), and liver-kidney yin-deficiency syndrome (LKYDS) are typical TCM syndromes for CHB diseases. They represent excess, excess-deficiency mingled, and deficiency TCM syndromes, respectively, which are widely used in disease diagnosis. However, the biological basis for the TCM syndromes remains unclear. Various studies have shown that the reductionist approach of Western medicine is not suitable for the scientific basis of TCM syndromes [11, 12]. TCM follows a traditional approach, focusing on theories and clinical symptoms, but lacks modern scientific explanation, while Western medicine focuses on detailed investigations with modern technologies at microscopic scales. By means of phenotype and systems biology, the holistic study of TCM has become a very popular research topic in modern science.

Currently, essence of different TCM syndromes in CHB was revealed by applying genomics, proteomics and metabonomics technics [13, 14]. Gene specification of syndromes demonstration and the change of gene expression in TCM syndromes has been investigated. For example, microarray test and RT-PCR were carried out in blood sample from LGHDS and LDSDS in CHB and liver cirrhosis and G-protein-coupled receptor protein-signaling pathway was found related to differentiating two TCM syndromes [15]. Epigenetic differences of CHB were also showed in different TCM syndromes [16]. Song et al. [17] performed surface-enhanced laser desorption ionization time-flight mass spectrometry (SELDI-TOF/MS) on two cohorts of CHB patients with excess or deficiency syndrome and found two significant serum proteins for classifying these syndromes. Adopting similar proteomics techniques, Liu et al. [18] also found four proteins differentially expressed in five typical TCM syndromes. Tongue coating is one of the important foundations of TCM syndrome signature. Zhao et al. [19, 20] demonstrated microbiota and metabolic difference of CHB patients with different TCM syndromes. Above all, biomolecules (including genes, proteins, and metabolites) expression and regulation rules were researched with the laws and methods in genomics, proteomics and metabonomics. Especially by comparing and analyzing the differences of biomolecules expression profile, specification and differentiation of TCM syndromes were proved. Our previous studies have also shown that several biomolecules may be potential markers for TCM syndrome differentiation in CHB [21–23]. Similarly, most potential biomarkers for TCM syndrome are selected from differentially expressed molecules [21, 24–26]. However, with the characteristics of “internal and external deficiency, dynamic space–time change, and multi-dimensional interface” [27], the complexity of dynamic changes mechanism of TCM syndromes have rarely been reported.

To address these issues, recent studies have provided novel strategies from different viewpoints. Among which, network perspectives and network-based approaches were approved as powerful and effective techniques in systems biology. Network-based methods are widely used in disease gene prediction [28, 29], drug discovery [30], and biomarker identification [31, 32]. Considering the complicated and dynamic characteristics of CHB-TCM syndromes, it is necessary to introduce a new process to integrate the interactive effects of individual molecules. Here, we introduce our statistical model for dynamic change: the dynamic network biomarker (DNB) method [33]. The DNB method can identify the critical state of TCM-syndrome progression, based on the correlation and fluctuation of molecules. Unlike the traditional molecular biomarker approach, the DNB method identifies differentially associated networks in a dynamic manner, to determine the corresponding functional DNBs of networks [34, 35].

In this present study, we proposed that dynamic changes in TCM syndromes fit the statistical model of the DNB method. Thus, we identified the critical transition of the CHB-TCM syndrome: changing from excess syndrome (LGDHS) to deficiency syndrome (LKYDS) at a network level. Moreover, we found that DNBs may play an important role in dynamic changes in the CHB-TCM syndrome.

## Methods

### Participants

A total of 244 blood samples that met the eligibility criteria were collected from Shuguang Hospital, Shanghai University of TCM. This study was approved by the Official Ethics Committee of the Shanghai University of TCM, and written informed consent was obtained from all participants. All healthy volunteers had no history of liver disease, no viral infection, and no other diseases. Sixty-four samples were used for microarray detection, including healthy group (n = 16), LGDHS (n = 16), LDSDS (n = 16), and LKYDS (n = 16). The TCM syndrome types were identified according to the guideline for the prevention and treatment of CHB, formulated by the Chinese Society of Hepatology and Chinese Society of Infectious Diseases, Chinese Medical Association [36]. All patients were diagnosed by attending TCM physicians at their first visit; and their diagnoses were confirmed by three chief TCM physicians, who are considered experts and usually have over 30 years of practice in TCM.

### RNA isolation and microarray detection

All blood samples were from the peripheral venous blood of patients with CHB and healthy donors. Apart from the 64 samples for microarray detection, another 57 samples of microarray data including LGDHS (n = 20), LDSDS (n = 20), and LKYDS (n = 17) were provided for validation. The samples were immediately frozen in liquid nitrogen and then stored at − 80 °C. The RNA was extracted using TRIzol^®^ Reagent (Invitrogen, Carlsbad, CA, USA) according to the manufacturer’s protocol. A quality control check was carried out using the NanoDrop ND-1000. The RNA concentration ranged from 1.5 to 12 ng/μL.

The total RNA profiles were extracted, and the biotinylated cDNA was hybridized to the Affymetrix U219 Whole Human Genome microarray (GeneTitan, Affymetrix, Santa Clara, California, USA). 60 ng of RNA was labeled and hybridized for each array. Hybridization signals were detected with the GeneChip Scanner 3000 (Affymetrix, Santa Clara, California, USA); the data were normalized using Gene Spring Software 11.0 (Agilent Technologies, CA, USA). All raw data were transformed into log 2 scale, and then, the expression levels were normalized to zero mean and unit sample variance.

### DNB analysis

Progression of both TCM syndromes and complex diseases usually have nonlinear transitions [33, 35, 37]. Such a transition plays key roles in biological processes. Identifying the critical transition in the progress is important for understanding the essence of TCM syndrome change. Thus, we introduced a mathematical model, i.e., the DNB method to identify the predictive biomarkers and understand the molecular mechanisms of TCM syndrome dynamic change. The DNB method has been applied successfully to studies in multiple fields (e.g., disease diagnosis and prognosis, therapeutic response, cell differentiation) [35, 38–40].

In nonlinear dynamical theory, there is a dominant group of molecules (i.e., DNBs), when a system is near the critical state. Using molecular fluctuation information (i.e., dynamical information) and network information (i.e., correlation information among molecules), DNBs could be identified when they satisfied three criteria [33].

1. The DNBs are highly fluctuated at the critical stage, with high coefficients of variation (*CV*).
2. The DNBs are highly correlated with each other in terms of their expression, and Pearson correlation coefficients (PCC) among them (*PCC*_*in*_) in absolute values is high.
3. The DNBs are weakly correlated with other molecules: the pearson correlation coefficients between them and other molecule clusters (*PCC*_*out*_) in absolute values is low.

In detail, the main steps are as follows:

- data preparation: genomic expression data of four stages (t = healthy, LGDHS, LDSDS and LKYDS, while healthy samples are considered as control). Let *CV* = 1, *PCC*_*in*_ = 1, *PCC*_*out*_ = 1 at healthy stage.
- Coefficient of variance test: select the genes with significant high CV basing on expression profile. The criterion is selecting genes with top 30 present CV value. Then we have a module of genes with high CV at t, M_t_C.
- Intra-correlation test: calculating average PCC of the genes from previous chosen M_t_C at each stage.
- Inter-correlation: calculating average PCC between inside and outside genes of M_t_C.
- DNB test: considering the above criteria, we used the following criterion index (CI) to quantify DNBs, as well as the tipping point.
  1 $$ {\text{CI}} = CV\frac{{PCC_{in} }}{{PCC_{out} }} $$
  where *CV* is the average coefficient of variance of the DNBs, *PCC*_*in*_ is the average PCC of the cluster of molecules in absolute values, and *PCC*_*out*_ is the average PCC between the cluster of molecules and other molecules in absolute values.
- Clearly, (1) represents the three conditions of the DNB. For each stage, the score of every module was calculated by the above formula and the best module with the highest score was regarded as the potential DNB in this stage. Then, these identified potential DNBs in each stage were compared with each other, and DNBs with the highest CI score in all time points was the DNBs for detecting the critical stage. The stage corresponding to the DNBs was so called critical stage.

### Functional analysis

Gene Ontology (GO) analysis and Kyoto Encyclopedia of Genes and Genomes (KEGG) pathway enrichment analysis were analyzed with clusterProfiler package of R [41, 42].

### Serum cytokine measurements

Serum samples from 25 LGDHS, 25 LDSDS, and 25LKYDS were collected by centrifugation (Model 3500; KUBOTA, Tokyo, Japan) at 5700×*g* for 10 min at 4 °C, aliquoted and stored at − 80 °C until analysis. A multiplex biometric enzyme-linked immunosorbent assay (ELISA)—containing dyed microspheres conjugated with a monoclonal antibody specific for a target protein—was used according to the manufacturer’s instructions. Soluble molecules were measured using two commercially available kits (BioPlex Assay: M50-0KCAF0Y and MF0-005KMII; Bio-Plex, Bio-Rad Laboratories Inc., Hercules, CA, USA). Forty-eight cytokines were assessed simultaneously using the Bio-Plex system: (i) 27-plex panel of IL-1β, IL-1rα, IL-2, IL-4, IL-5, IL-6, IL-7, IL-8, IL-9, IL-10, IL-12p70, IL-13, IL-15, IL-17, eotaxin, FGF basic, G-CSF, GM-CSF, IFN-γ, IP-10, MCP-1, MIP-1α, MIP-1β, PDGF-ββ, RANTES, TNF-α, and VEGF; and (ii) 21-plex panel of IL-1α, IL-2Rα, IL-3, IL-12p40, IL-16, IL-18, CTACK, GROα, CXCL9, SDF-1α, HGF, IFNα2, LIF, MCP-3, M-CSF, MIF, β-NGF, SCF, SCGF-β, TNF-β, and TRAIL. These two kits covered the range of all the cytokines potentially involved in the pathophysiology of liver cirrhosis. The selection of specific cytokines in the study was based on previously available reports on liver disease [43, 44]. Assays were performed in duplicate by following the standard operating protocol provided by the Bio-Plex Multiplex cytokine assay. Serum levels of all proteins were determined using a Bio-Plex array reader (Luminex, Austin, TX) that quantified multiplex immunoassays in a 96-well plate with very small fluid volumes. The analyte concentration was calculated using a standard curve, with software provided by the manufacturer (Bio-Plex Manager Software, Bio-Rad Laboratories, Inc., CA, USA). The limit of detection (mean negative control plus 3× standard deviation) was determined for each assay. A Millipore xMAP Kit (HCYTOMAG-60K-06; Merck Millipore, Billerica, MA, USA) was applied to detect the serum levels of IL-9, IL-2Rα, and GM-CSF in another independent cohort of patients for validation. Each experiment was performed according to the manufacturer’s protocol [45]. The cytokine concentrations were calculated using a standard curve with the software provided by the manufacturer.

### iTRAQ, protein identification, and relative quantification

Forty-eight samples—patients with CHB (12 LGHDS, 12 LDSDS, and 12LKYDS) and 12 healthy participants—were subjected to isobaric tags for relative and absolute quantitation (iTRAQ) analyses, among which, an equal amount of 10 different samples were mixed to produce a sample pool. Thus, each TCM syndrome group had three biological replicates. A total of 100 μg protein from each group was centrifuged. The samples were then digested with trypsin at 37 °C, overnight. Samples were labelled with the iTRAQ reagents (AB SCIEX, Foster City, CA, USA) and fractionated by strong cation exchange (SCX), according to the manufacturer’s instructions and as previously described [46, 47]. All labeled peptides were mixed and analyzed by liquid chromatography coupled with tandem mass spectrometry (LC–MS/MS). Protein identification and quantification were performed using the Maxquant 1.3.0.5. The LC–MS/MS data were searched against uniprot human (136,615 sequences, downloaded on August 2, 2014). The ratio of protein expression between the two groups (<0.8 or >1.25) was considered significant.

### Data processing

Differences in gene expression levels between groups were compared using the Mann–Whitney U test. The two-tailed *p *<0.05 was considered statistically significant. All networks were visualized by Cytoscape software 3.3.0 (http://www.cytoscape.org/). Receiver operating characteristic (ROC) curve was established to assess the diagnostic ability. All data analyses were performed using the statistical software R version 3.1.0 (http://www.R-project.org).

## Results

### General information of patients and differentially expressed genes of CHB-TCM syndromes

**Fig. 1:**
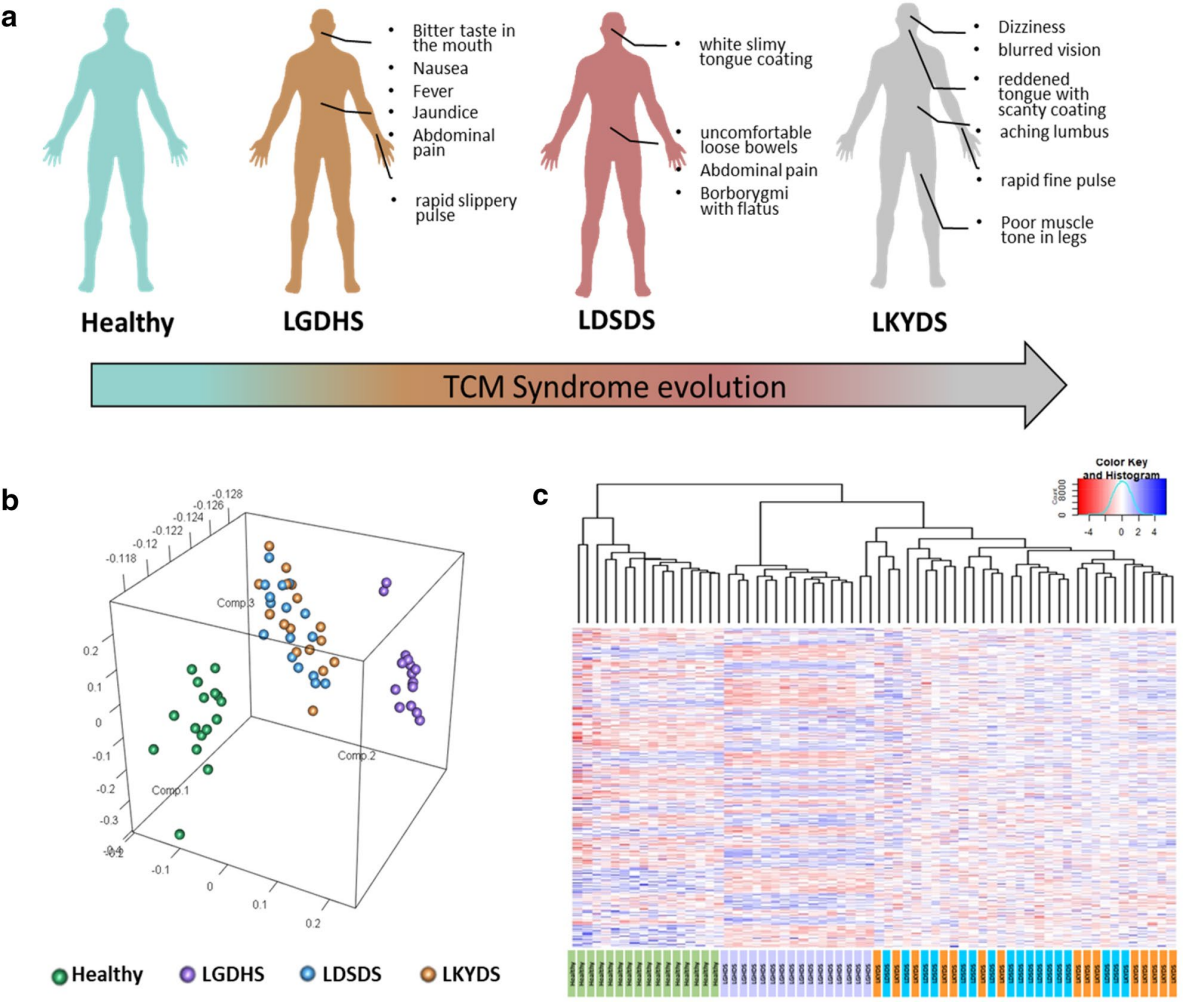
The progression of traditional Chinese medicine (TCM) syndrome in chronic hepatitis B (CHB) and gene expression analyses. **a** A schematic diagram illustrates the progression of TCM syndromes, each typical TCM syndrome has a different clinical manifestation. **b** Three-dimensional principal component analysis (PCA) shows clustering of 60 samples with TCM-syndrome progression. Each small spot represents the principal component score of the top three principle components for each sample. **c** Unsupervised hierarchical clustering of 60 samples based on 4252 differentially expressed genes (DEGs). Similar to (**b**), the healthy and LGDHS groups were clustered together, while the LDSDS were mingled with LKYDS. **b**, **c** Green, healthy; purple, LGDHS; blue, LDSDS; orange, LKYDS

A total of 244 participants were enrolled in this study, including 73 with LGDHS, 73 LDSDS, 70 LKYDS, and 28 healthy volunteers (Additional file 1: Table S1). The distribution of liver function indexes showed no significant difference in patients with the three typical TCM syndromes (data did not show). In TCM, they represent excess, excess-deficiency mingled, and deficiency TCM syndromes, respectively (Fig. 1a). To detect the dynamic network changes in patients, we conducted gene expression measurements from 48 patients with CHB including 16 LGDHS, 16 LDSDS, and 16 LKYDS, and 16 healthy participants. After gene expression profiles, a three-dimensional principal component analysis (PCA) was performed to compare their gene expression profiles (Fig. 1b). Clearly, the healthy and LGDHS groups were clustered together, while the LDSDS were mingled with LKYDS, suggesting LDSDS and LKYDS may represent the late stage and they different from early stage of TCM syndrome, such as healthy and LGDHS group. To further analyze the correlation between the gene expression profiles and TCM syndromes, an unsupervised hierarchical clustering was performed by PCC distance based on 4252 differentially expressed genes (DEGs) (Fig. 1c). The first branch allowed the separation of the healthy group from the CHB-TCM syndromes. A sub-branch partitioned the LGDHS and the other two TCM syndromes. The LDSDS and LKYDS groups were mingled with each other. The result of the hierarchical clustering was consistent with the PCA, which also implied that LGHDS was special and different from the other stages, as the early stage of TCM-syndrome evolution.

### DNB analysis identifies the phase transition for TCM syndrome changes from excess to deficiency

**Fig. 2:**
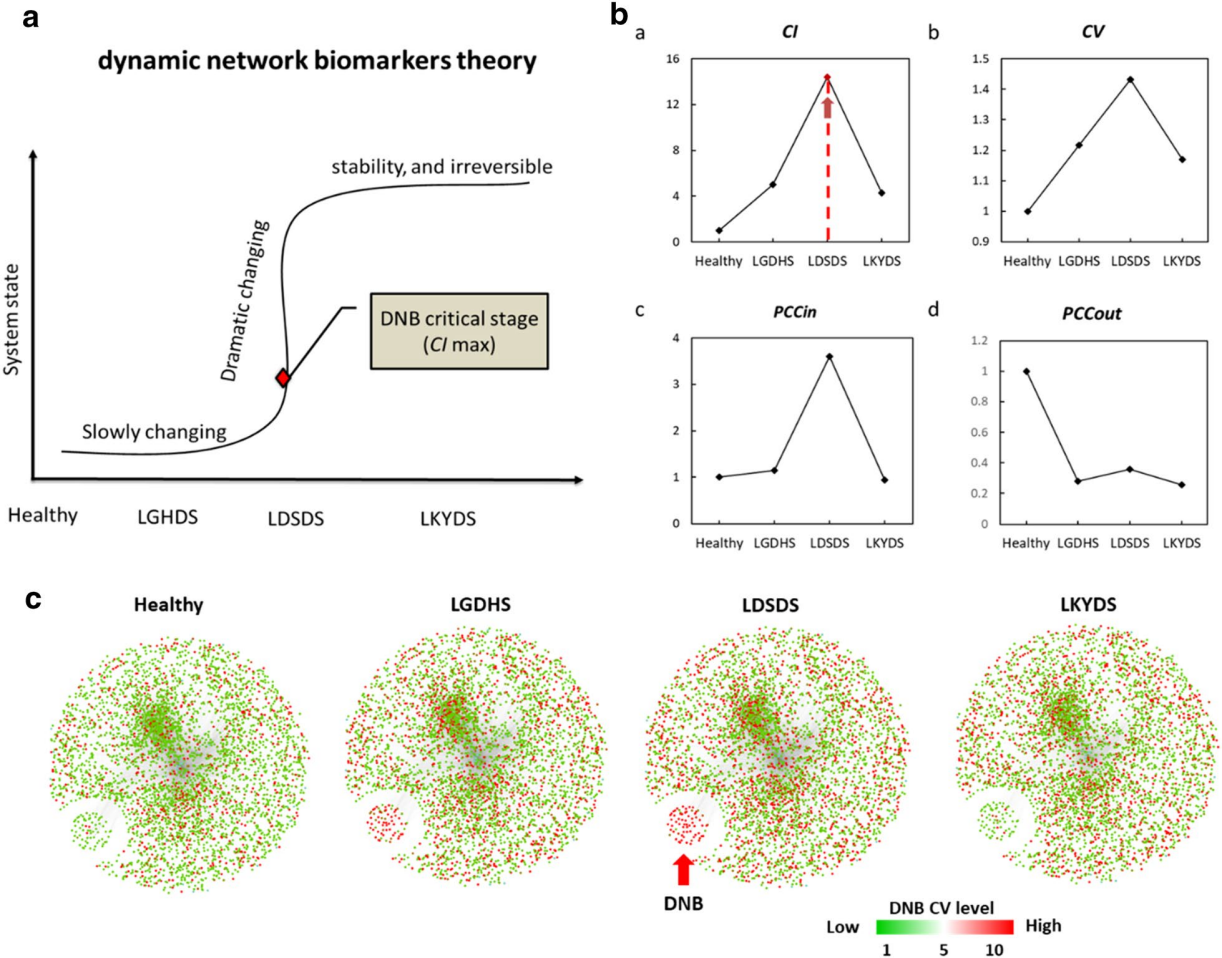
A brief model of dynamic network biomarkers (DNB) theory and DNB analysis results. **a** Liver-gallbladder dampness-heat syndrome (LGDHS) (excess TCM syndrome) usually happens at disease onset or TCM-syndrome change. After the tipping point, the system drastically deteriorates to weakness, for instance, liver-kidney yin-deficiency syndrome (LKYDS) (deficiency TCM syndrome). The DNB method can identify the dramatic changing state by analyzing molecular fluctuations at each stage. **b** These four diagrams visually show the three key criteria of DNBs over four different stages during TCM-syndrome progression. *CV* is the average coefficient of variance of the DNBs, *PCC*_*in*_ is the average Pearson correlation coefficient (PCC) of the cluster of molecules in absolute values, and *PCC*_*out*_ is the average PCC between the cluster of molecules and other molecules in absolute values, after comparing with the corresponding controls. CIs were calculated according to the DNB formula (method) to seek the system tipping point. After calculation, LDSDS was recognized as the critical stage of TCM-syndrome progression. **c** Series of illustrations of dynamic changes in the network structure. Node color reflects the CV of the corresponding molecule. Clearly, DNBs are strongly correlated and fluctuated at the LDSDS stage

Identifying the critical stage of TCM-syndrome change is crucial to diagnose TCM syndromes. Our DNB model was developed to detect this critical change by measuring fluctuations and correlations of molecules (Fig. 2a). The results of the three criteria for DNBs are shown in Fig. 2b. At the critical stage, (i) the expressions of DNBs become highly fluctuated (high coefficient of variation); (ii) DNBs are highly correlated (PCCin are high), and (iii) correlations between DNBs and non-DNBs are weak (PCCs are low). Three conditions above can be explained by following sentences: from the observed data, the appearance of a group of genes with strongly collective fluctuations when the system is near the critical state. Thus, these genes are the predictive biomarkers for this critical transition. Usually, it is irreversible. When CI reaches a peak or increases markedly during the measured periods, the biological system is at the critical period or tipping point [48]. Based on these criteria, we found that the critical stage of TCM-syndrome change—from excess to deficiency—is LDSDS. Accordingly, 52 genes were identified as DNBs (Additional file 1: Table S2). Functional analysis showed that DNBs were enriched in the regulation of fibrinolysis and complement and coagulation cascade pathways (Additional file 1: Table S3), which are important processes in extracellular matrix (ECM) formation and immune function. Then, we measured the performance of candidate DNBs at four stages and the scores of the candidate DNBs were summarized in the CI (Fig. 2b). Clearly, the CI of the candidate DNBs at LDSDS was significantly higher than that of the other three stages, so these candidate DNBs signaled drastic deterioration during TCM-syndrome progression. We then constructed a series of networks with PCC of gene-pairs, to illustrate the corresponding dynamic change in the network structure and expression variations in the identified DNBs and their neighbor molecules (Fig. 2c). As shown in Fig. 2c, at the LDSDS stage, the DNBs have strong fluctuations compared with the other three stages; and the links between DNBs and other molecules are significantly greater, which indicated drastic changes in co-expression relationships within DNBs, or between them and other molecules, when the biological system approached the critical stage.

### DNB-DEG associated network is flipped before and after the critical transition

**Fig. 3:**
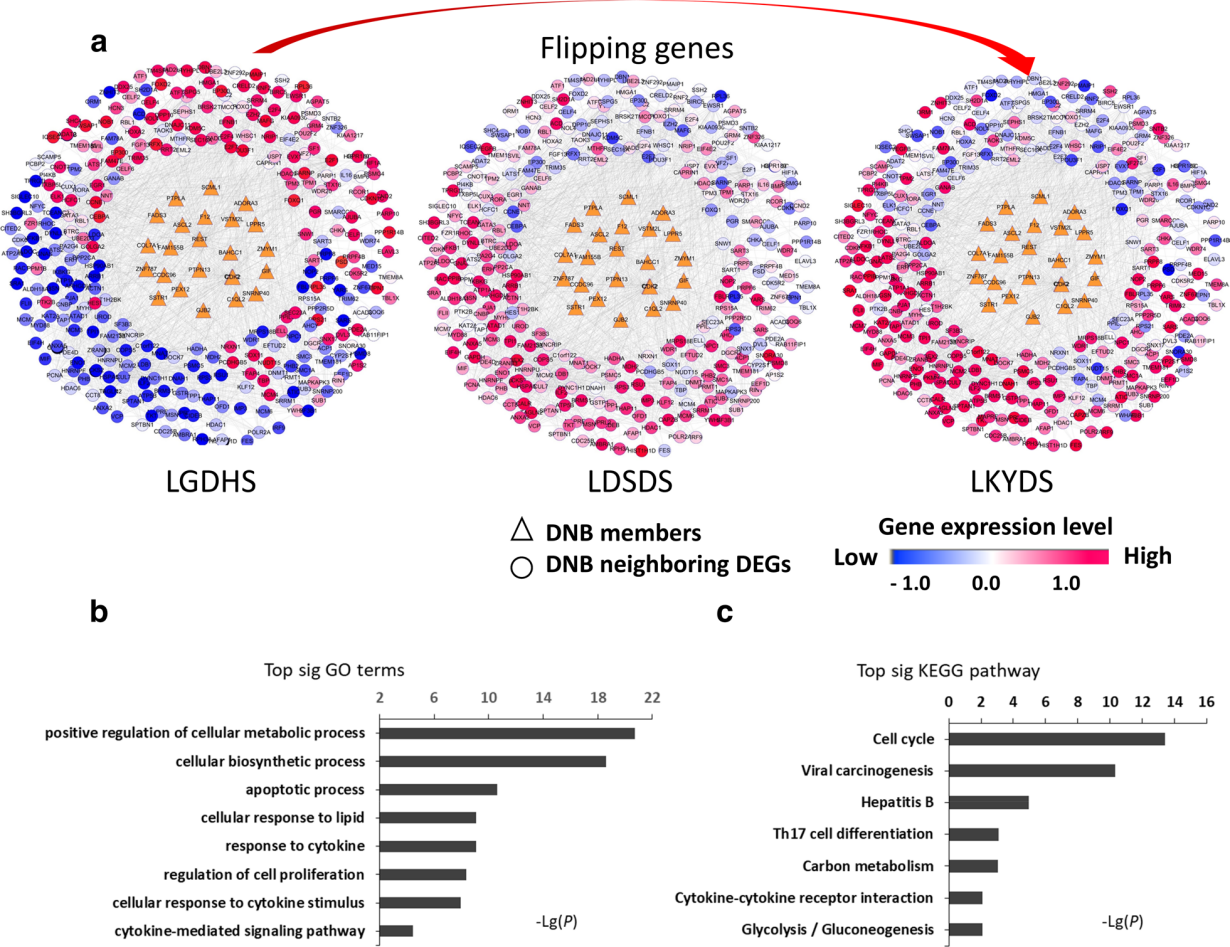
Dynamic network biomarker–differentially expressed gene (DNB–DEG) network, before and after the critical stage. **a** Illustrations of dynamic change in the expressions of the DNB-associated network before and after the critical stage (LDSDS). **b**–**c** Functional analyses of the flipped DEGs

DNBs are a group of molecules with strong correlations and fluctuations at the critical stage, which are different from DEGs. DNBs regulate DEGs before and after the critical stage; thus, they may perform biological functions in TCM-syndrome change. To study the mechanism of the drastic deteriorations during changes in the TCM syndrome, we constructed the DNB-DEG-associated network including 332 DNBs and DEGs (Additional file 1: Table S4), by integrating DNBs and their first-order neighboring DEGs according to the whole human molecular network. Two-hundred-eight DEGs—from low (or high) at the LGDHS stage to high (or low) at the LKYDS stage—were directly linked to 24 DNBs (Fig. 3a).

Functional analyses were performed to describe the function change induced by the flipped DEGs (Additional file 1: Table S5), which may be regulated by DNBs. GO analysis showed the flipped DEGs were enriched in metabolic processes, apoptosis, and especially in cytokine-related pathways, including response to cytokine, cellular response to cytokine stimulus, and the cytokine-mediated signaling pathway (Fig. 3b). KEGG pathway enrichment of flipped DEGs also showed similar results: they enriched the pathway of cytokine–cytokine receptor interaction (Fig. 3c). Hepatic inflammation is a common trigger of liver disease and it should be considered from the perspective of chronic inflammation [49]. There is increasing evidence that several cytokines mediate hepatic inflammation, apoptosis and necrosis of liver cells, cholestasis, and fibrosis [50, 51]. These results indicated that cytokine action plays a key role in TCM-syndrome change.

### Dynamics of functional phenotypes influenced by the DNB-associated network before and after the critical transition

**Fig. 4:**
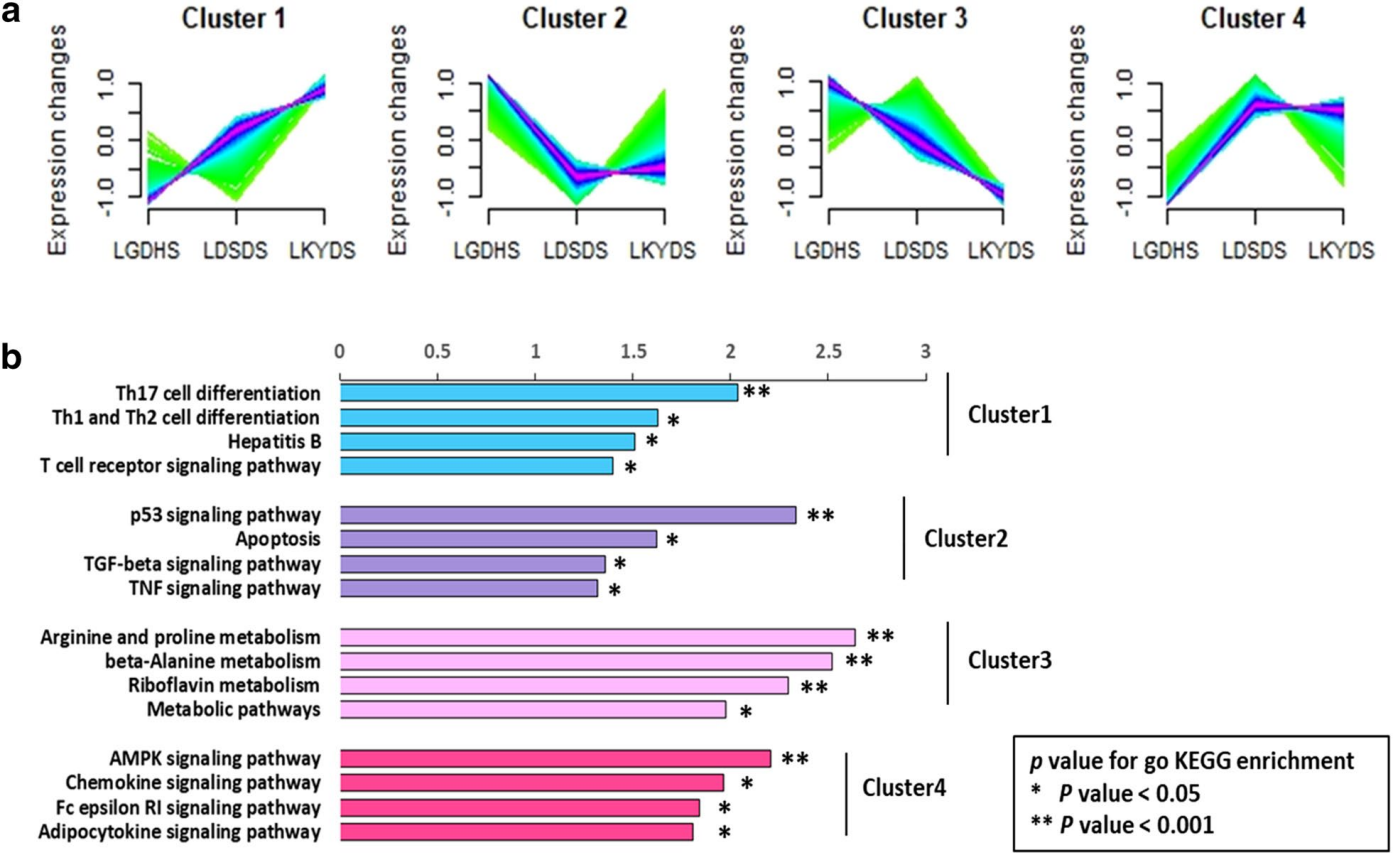
Functional phenotyping of dynamic network biomarkers (DNBs) and differentially expressed genes (DEGs) in a DNB-associated network. **a** Four dynamic expression patterns of DNBs and DNB-related DEGs were identified by the Mfuzz clustering method. **b** The bar graph shows related Kyoto Encyclopedia of Genes and Genomes (KEGG) pathways, which were enriched according to corresponding DNBs and DNB-related DEGs in different dynamic patterns

To further understand how DNBs are involved in TCM-syndrome change, functional analyses on the dynamic patterns of DNBs and DNB-associated DEGs, before and after the critical stage, were performed. Firstly, DNBs and DNB-associated DEGs were classified by Mfuzz [52] into four clusters (Fig. 4a): members of Clusters 1 and 3 were upregulated before the critical stage, while members of Clusters 2 and 4 were upregulated after the critical stage. Thus, DEGs in Clusters 2 and 4 may be influenced by their associated DNBs earlier than those within Clusters 1 and 2 (Fig. 4a).

We then performed the functional analyses of members in each cluster (Additional file 1: Table S6). Among which, 16 liver disease related pathways were chosen and are listed in Fig. 4b. The inflammation activities-related pathway was mainly affected by DNBs in Cluster 1. Meanwhile, pathways involving cell apoptosis and growth inhibition were mainly related to members of Cluster 2, including the P53 signaling pathway and TGF-β signaling pathways. After the critical stage, metabolism-related pathways were significantly affected by members of Cluster 3, while the expression of related members in Cluster 3 increased before the critical stage and then decreased. In Cluster 4, molecules were enriched in chemokine, and adipocytokine signaling pathways. Expression of related members in Cluster 4 decreased before the critical stage and increased after.

These results concur with previous reports: inflammation plays a key role in liver disease progression. Patients’ conditions are usually aggravated from excess to deficient TCM syndrome [53].

### Validation of differentially expressed cytokines regulated by DNBs

Previously, we found that DNB-regulated flipped DEGs were related to cytokine pathways. We performed cytokine profiling to validate the differences in cytokine expression between different TCM syndromes. Serum cytokines from 25 LGDHSs, 25 LDSDSs, and 25 LKYDSs were determined by ELISA-based cytokine profiling.

**Fig. 5:**
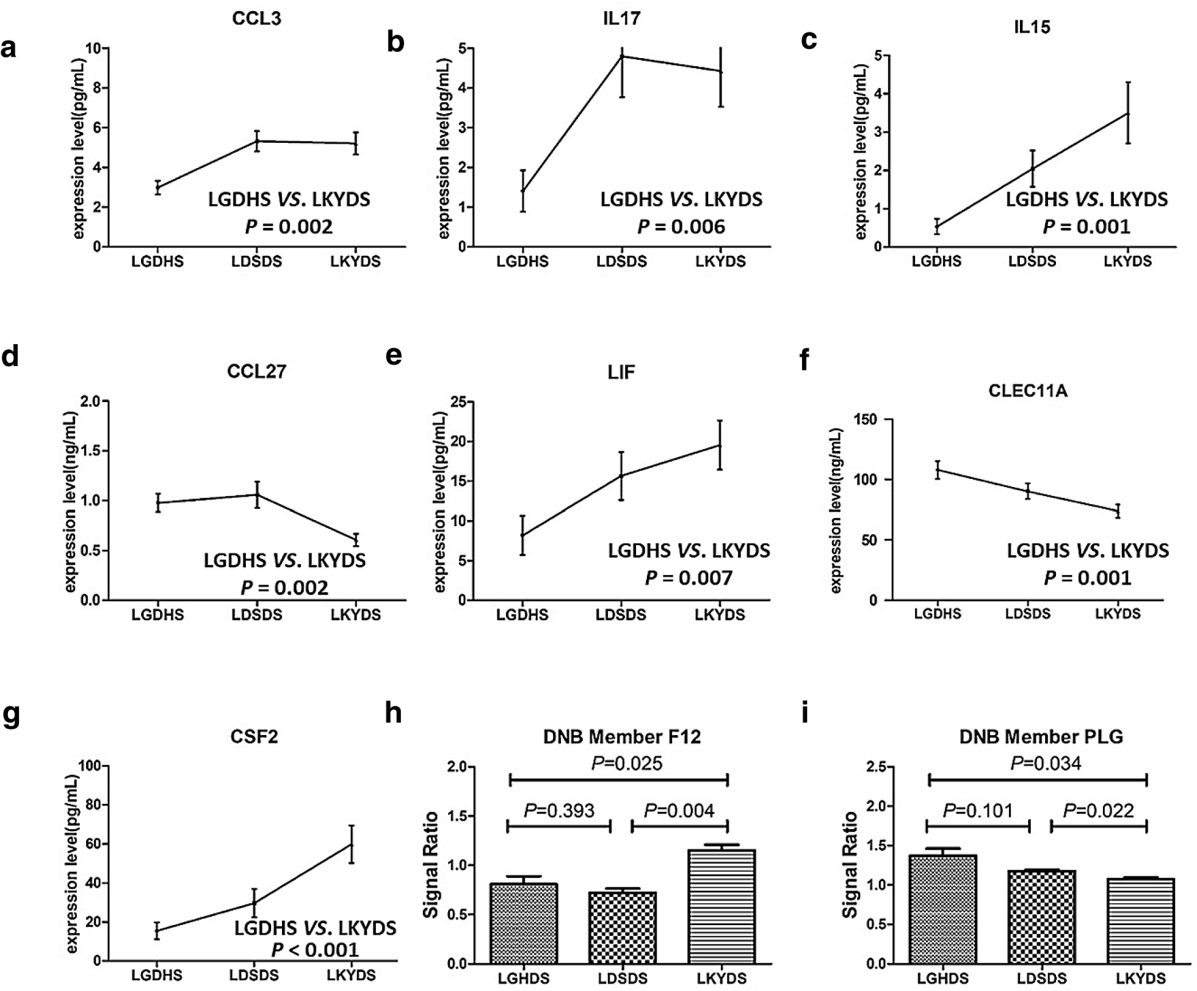
Expression of seven cytokines changed significantly from liver-gallbladder dampness-heat syndrome (LGDHS) to liver-kidney yin-deficiency syndrome (LKYDS). **a**–**g** Cytokine expression of CCL3, IL17, IL15, CCL27, LIF, CLEC11A, and CSF2. These cytokines were significantly expressed between LGHDS and LKYDS. The vertical axis represents absolute quantification of cytokines measured by enzyme-linked immunosorbent assay (ELISA). **h**, **i** Validation of DNBs plasminogen (PLG) and coagulation factor XII (F12) in the proteomic data. The vertical axis represents signal ratio compared to the healthy group. **a**–**i** The *p* values were measured by Mann–Whitney U tests

The results showed that 7 cytokines were significantly expressed (*p* < 0.01) between LGDHS and LKYDS (Fig. 5). This is consistent with previous studies, that during the development of TCM syndrome evolution, cytokines, chemokines, and growth factors play important roles in viral clearance, infection control, inflammation, regeneration, and fibrosis in CHB [24]. From excess TCM syndrome (LGDHS) to deficient TCM syndrome (LKYDS), the TCM syndrome evolves over time with liver damage in CHB.

Furthermore, to validate the expression pattern of DNBs, we applied iTRAQ to obtain the proteomic data of different TCM syndromes. Samples from 12 LGDHS, 12 LDSDS, 12 LKYDS, and 12 healthy participants were collected to acquire proteomic data by iTRAQ. DNBs are a cohort of molecules with strong correlations and fluctuations. The results from iTRAQ showed that plasminogen (PLG) and coagulation factor XII (F12), which belong to DNBs, were significantly expressed during TCM-syndrome progression from LGDHS to LKYDS.

### Diagnostic ability assessment for PLG and F12

**Fig. 6:**
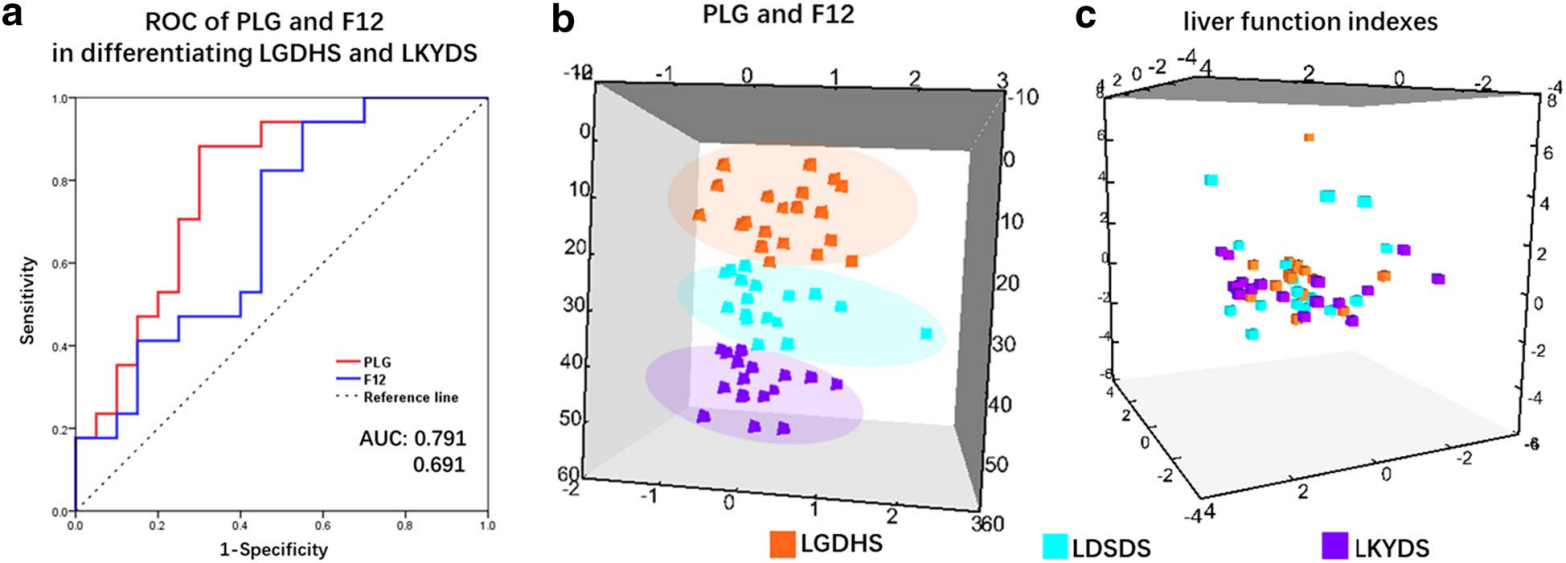
Diagnostic ability assessment for PLG, F12, and liver function indexes. **a** Receiver operating characteristic (ROC) ROC curve analysis of PLG and F12. AUC of PLG = 0.795 (95% CI 0.644 to 0.938), AUC of F12 = 0.795 (95% CI 0.520 to 0.862). **b** PCA of 57 samples including 20 LGDHS, 20 LDSDS and 17 LKYDS in an independent cohort by PLG and F12. **c** PCA of samples by liver function indexes (i.e. GGT, ALB, TBIL, DBIL, GLO, AST, ALT, TP, and ALP). Purple, LGDHS; orange, LKYDS

In clinical practice, biomarkers are expected to provide accurate predictions. We used receiver operating characteristic (ROC) ROC analysis and PCA to assess the diagnostic ability of DNB member PLG and F12 for stages before and after critical stage, which is LGHDS and LKYDS. We found PLG and F12 reached high diagnostic ability with AUC = 0.791 and 0.691 (Fig. 6a). Similarly, PLG and F12 clearly separate three TCM syndromes groups by PCA (Fig. 6b). By contrast, PCA was also applied on samples by liver function indexes, (i.e. AST, ALT, GGT, ALB, TBIL, DBIL, TP, GLO and ALP). Clearly, liver function indexes were unable to separate three TCM syndromes (Fig. 6c). These results suggest that, PLG or F12 rather than liver function indexes improves the clinic diagnosis and prognosis.

## Discussion

TCM is widely used to treat CHB disease, with an advantage in early intervention and combined therapies [26]. However, TCM syndromes lack objective assessment and heavily rely on physician experience. In TCM theory, with the progression of disease, the TCM syndrome also changes. The struggle between “healthy qi” and “pathogenic qi” refers to disease occurrence and development, which concurs with the process of inflammation in Western medicine. Thus, TCM syndromes dynamically changed as CHB progressed. The etiology and pathogenesis of CHB are complex, showing various types of TCM syndromes in the clinic. According to the different clinical manifestations of patients with CHB, different TCM syndromes appeared at different stages in the progression of CHB. Discovering DNBs and the mechanism of dynamic changes in TCM syndromes are key issues in TCM research.

As a model-free approach, the DNB method effectively identifies the critical stage during disease/syndrome progression, basing on multiple samples in each stage. In this study regarding CHB-TCM-syndrome changes, we identified LDSDS as the critical stage from healthy to LKYDS. By integrating DNBs and their first-order DEGs per the molecular network of the whole human, we found that many DEGs showed inverse expressions from high (low) to low (high) before and after the critical stage. Functional analysis of DNBs and their regulated molecules indicated that cytokine action was involved in the dynamic changes in the TCM syndrome. This was consistent with reports that persistent infection of HBV in vivo is determined by virus and immune function. The imbalance of Th1/Th2 cells and their cytokines is an important cause of CHB [54]. Results have shown that cytokines were significantly differentially expressed between TCM syndromes. As the TCM syndrome changes, the immune response may be more intense from LGDHS to LKYDS. The degree at which inflammation causes tissue injury changes with the body’s immune response, which is reflected in cytokine expression.

Among the 17 significantly differentially expressed cytokines, several have been shown to be associated with TCM syndromes [53, 55–57]. For instance, IL-18 was found to be significantly expressed in LGHDS and LDSDS in patients with liver cirrhosis, by statistics analysis [56].

The effect of DNBs on TCM syndromes depended not on their differential expressions, but on collective fluctuations according to DNB theory. The relationship between DNBs and DEGs during critical transition at a network level was detected (Figs. 3, 6). The DNB-regulated DEGs, before and after the tipping point, were significantly enriched in the cytokine-mediated signaling pathway. Protein plays a crucial role in this function; thus, we further validated DNB expression in the proteomic data. In the DNBs, we found PLG and F12 presented significantly different expressions in the iTRAQ data. F12 is a plasma protease that, in its active form (FXIIa), initiates the procoagulant and proinflammatory contact system [58]. Recently, significant evidence has emerged, implicating F12 as a disease target of human thrombotic and inflammation diseases [59]. Coagulation factors play an important role in tissue repair and inflammatory responses. ECM deposition is the main pathological feature of hepatic fibrosis during the change from CHB to liver cirrhosis, even liver cancer. The fibrinolysis system plays an important role in ECM deposition. PLG is a plasma protein produced mainly by hepatocytes, which can be activated by plasminogen activator (PA), and then degrades many ECM components [60]. In our study, PLG exhibited the greatest attenuation in liver injury, which is consistent with a previous study [61]. The alternation of PLG is a sign of liver injury. Furthermore, we compare PLG and F12 with clinical data in CHB patients with LGDHS, LDSDS and LKYDS in the diagnostic ability for the TCM syndrome differentiation. We found PLG and F12 can clearly separate LGDHS LDSDS and LKYDS. However, the clinical data could not separate three TCM syndromes. This result indicated a potential value of clinical application of DNBs for the auxiliary diagnosis of TCM syndrome in CHB. PLG and F12 play an important role in initiating the progression of liver disease. Our DNBs findings and clinical significance should be further studied using a larger sample size in future.

## Conclusions

In this study, contrary to traditional molecular biomarkers, we demonstrated that the DNBs of TCM syndromes change in patients with CHB. Specifically, we found the tipping point of this change occurs at the LDSDS stage. During this process, the cytokine system was involved all the time. Therefore, DNBs can be used to analyze the molecular mechanism of TCM-syndrome progression, based on the identified leading networks. The DNBs PLG and F12 were confirmed to significantly change during TCM-syndrome progression and indicated a potential value of DNBs in auxiliary diagnosis of TCM syndrome in CHB.

## Supplementary information

**Additional file 1: Table S1.** Clinical characteristics of participants. **Table S2.** Detailed DNB members at tipping point. **Table S3.** Functional enrichment of GO biological processes and KEGG pathways for the identified DNB. **Table S4.** 322 DNB and DEG in DNB-DEG associated network. **Table S5.** Functional enrichment of GO biological processes and KEGG. **Table S6.** Functional enrichment of KEGG pathways for genes of each cluster.

## Data Availability

All the data used to support the findings of this study are available from the corresponding author upon reasonable request.

## Abbreviations

TCM: Traditional Chinese Medicine
DNB: dynamic network biomarker
LGDHS: the dampness-heat accumulation syndrome
LDSDS: liver-depression spleen-deficiency syndrome
LKYDS: the liver kidney Yin deficiency syndrome
CHB: chronic hepatitis B
CI: criterion index
iTRAQ: isobaric tags for relative and absolute quantitation
ROC: receiver operating characteristic
PCA: principal component analysis
